# The Influence of Everyday Acoustic Environments on the Challenging Behavior in Dementia: A Participatory Observation Study in Nursing Homes

**DOI:** 10.3390/ijerph20054191

**Published:** 2023-02-26

**Authors:** Arezoo Talebzadeh, Ine Decoutere, Tara Vander Mynsbrugge, Dick Botteldooren, Paul Devos, Francesco Aletta, Dominique Van de Velde, Patricia De Vriendt

**Affiliations:** 1 Department of Information Technology, WAVES—iGent, Ghent University, 9000 Ghent, Belgium; 2 Department of Rehabilitation Sciences, Faculty of Medicine and Health Care Sciences, Ghent University, 9000 Ghent, Belgium; 3 Occupational Therapy Department, Artevelde University of Applied Sciences, 9000 Ghent, Belgium; 4 Institute for Environmental Design and Engineering, The Bartlett, University College London, London WC1H 0NN, UK; 5 Frailty in Ageing (FRIA) Research Group, Department of Gerontology and Mental Health and Wellbeing (MENT) Research Group, Vrije Universiteit, 1050 Brussel, Belgium

**Keywords:** acoustics, soundscape, acoustical triggers, behavioral and psychological symptoms of dementia (BPSD), challenging behavior (CB), Alzheimer, dementia, sound environment, auditory stimuli

## Abstract

Challenging behavior (CB) is a group of behaviors, reactions and symptoms due to dementia, which can be challenging for the caregivers. The study aims to research the influence of acoustics on CB in people with dementia (PwD). An ethnographic method was used to study the daily life of PwD in their nursing homes with a specific focus on how people react to everyday environmental sounds. Thirty-five residents were included in the sample based on purposeful, homogeneous group characteristics and sampling. Empirical data were collected using 24/7 participatory observations. The collected data were analyzed using a phenomenological–hermeneutical method: a naïve understanding, a structural analysis and a comprehensive understanding. The result shows that the onset of CB depends on whether the resident feels safe and is triggered by an excess or lack of stimuli. The excess or shortage of stimuli and whether and when it affects a person is personal. It depends on various factors, the person’s state and the time of day, the nature of the stimuli, familiarity, or strangeness is also a determining factor for the onset and progression of CB. The results can form an essential basis for developing soundscapes to make the PwD feel safe and reduce CB.

## 1. Introduction

The growing ageing population is accompanied by more complex and chronic health issues such as dementia. Every 20 years, the number of people with dementia (PwD) will double; reaching 131.5 million worldwide by 2050 [1]. 

Dementia is a progressive illness characterized by cognitive and functional decline. This decline often co-occurs with a change in behavior, such as restlessness and aggression. This behavior is mainly referred to as behavioral and psychological symptoms of dementia (BPSD) [2,3]. However, the term ‘challenging behavior’ (CB) [4] is correct as it is less stigmatizing. CB refers to the challenges for the informal and professional caregivers to deal with this behavior, while BPSD describes the behavioral symptoms from a biomedical perspective. 

CB represents a heterogeneous group of challenging behaviors, reactions and symptoms, which is frequent in PwD [5]. A total of 90% of individuals with Alzheimer’s exhibit at least one CB and about one-third experiences serious problems [6,7]. Overall, CB has several consequences in different domains. Previous studies have shown that CB significantly contributes to the overall cost of healthcare [8]. It is an essential factor for an inadequate prognosis, rapid rate of cognitive decline, increased hospitalization and urgent care visits, early institutionalization in nursing homes (NHs) and excessive disability [2,9]. Moreover, CB significantly impacts on the quality of life (QoL) of PwD and their healthcare provider [10,11,12,13].

Currently, there is no cure for dementia, and symptoms such as CB are addressed by available treatments [8]. Dementia guidelines recommend first treating CB non-pharmacologically. If non-pharmacological interventions demonstrate little or no result, pharmacological interventions can be carried out [14]. In practice, however, pharmacology is often the first choice of approach [3]. The number of PwD using antipsychotics is estimated to be between 19% and 46% in European NHs. Its use, however, is controversial because of the potential benefits being overshadowed by the potential harm [7]. 

Consistent data that support the use of non-pharmacological treatment for CB are lacking [15]. Many reviews report mixed results with little to no consistency of evidence to recommend or reject an intervention. A systematic review (SR) by Abraha et al. [16] examined nineteen non-pharmacological interventions to treat CB for PwD, including three environment-based studies. The overall conclusion of this SR showed that from 19 interventions, music therapy and behavioral management techniques (BMT) were effective in reducing CB. Functional analysis (FA) is part of the BMT. This behavioral intervention requires the therapist to look for the underlying function, meaning or problem causing the person’s distressing behavior [14]. Dyer and colleagues [17] stated that FA-based interventions were the first choice. 

With the growing evidence of non-pharmacological interventions, there is an increasing interest in adapting the environment [18], specifically the sonic environment, which is already a topic of many investigations in schools, restaurants and parks [19,20]. Brown et al. [21] described the role of sound in clinical environments and the damaging effects of sound, emphasizing mental health care. Andriga and Lanser [22] looked at sound and its impact on people’s behavior and QoL. Both concluded that excessive unwanted noise is harmful while quiet and pleasant sounds promote health. 

In healthcare, soundscapes—emphasizing people’s relationship to the sonic environments, whether natural, musical or synthesized—are increasingly used to reduce adverse consequences and improve positive effects [23]. Soundscape is defined as the “acoustic environment as perceived, experienced or understood by people in context” [24]. Soundscapes usually encompass different sounds that occur simultaneously or consequently [25]. Even though the interest in the perceived quality of indoor soundscapes is growing [26], currently, only a few studies are focusing on the effect of soundscapes (either perceived or objectively measured) on CB, especially within the context of NHs [26,27,28]. Soundscapes might positively influence CB since CB has a neurological basis, making the PwD more vulnerable to environmental, physical and psychological factors [5,7,29,30]. De Pessemier et al. [31] looked at the positive impact of personalized soundscapes for PwD. Kosters and colleagues [32] obtained promising results using apps and other IT-related infrastructures. The previous study by the research team focused on monitoring sound levels and the soundscape quality of nursing homes [26] and soundscape design for the management of BPSD in nursing homes [28,29,33]. This paper focuses on the development of BPSD in dementia in relation to acoustics in the context of nursing homes.

This study investigates the effect of the everyday soundscape of nursing homes on challenging behavior in people with dementia. 

Understanding the effect of the sonic environment on the Challenging Behavior of people with dementia will help design a better acoustical environment in nursing homes. Unfortunately, soundscape is not a common design standard, and usually, attention goes to eliminating mechanical noise with less attention to the day-to-day soundscape of nursing homes. The lack of studies emphasizing the importance of soundscape in nursing homes and the effect of sound on the challenging behavior of people with dementia is apparent. Understanding the relationship between different sounds and behavior change helps to improve the quality of life for PwD in NHs.

This research explores the factors in the onset and progression of CB in PwD living in NHs. Further research can be used to develop a valid model for enhancing QoL and modifying behavior in PwD through soundscapes.

This paper explains an ethnographic design method to observe the participants for a total of 420 h, followed by a phenomenological–hermeneutical analysis method which resulted in 152 meaning units. The structural analysis of these units then resulted in a theoretical model showing the relation between a person’s capacity to interpret sounds and the capacity to react to them.

## 2. Materials and Methods

### 2.1. Context of the Study

This study is part of a larger study (the AcustiCare project) in the Belgian Context in which we aim to improve the acoustic environment in nursing homes and to decrease the level of BPSD in persons with dementia by using adapted soundscapes. Therefore, we included different nursing homes. The acoustic environments in the various nursing homes were not statistically different regarding the sound level and loudness recorded between different days of the week, living rooms and time slots. In addition, everyday activities (and the sound levels they generate) occurred more or less consistently with the same intensity and over the same periods, regardless of the day, in all the nursing homes [26]. The distribution of sound levels across the NHs observed in the previous study was between 45 and 50 dBA (see Thomas et al., 2020) [28].

### 2.2. Research Design

An ethnographic method was used to study the daily life of PwD with a specific focus on how people react to day-to-day sounds in their NH. Ethnography is a qualitative method for collecting data often used in the social and behavioral sciences. Ethnographers observe life as it happens instead of trying to manipulate it in a lab. Data are collected through observations and interviews, which are then used to draw conclusions about how societies and individuals function. The PwD’s experiences regarding sounds were observed, promoting a comprehensive understanding of their experiences and behavior.

### 2.3. Sampling

The data collection included two waves of NHs. The NHs were selected based on convenience sampling [34], those with private rooms, a dining room, a sitting area and a cafeteria and known from the previous studies sharing a similar acoustic environment (see Section 2.1).The NHs had to be part of Flanders, the Dutch-speaking part of Belgium, and have a (para)medical and nursing team as imposed by the Flemish government [35]. PwD were selected in consultation with the head of each NH based on purposeful, homogeneous group characteristics’ sampling [36]. Inclusion criteria were PwD, assessed with the Mini-Mental State Examination (MMSE), living in a department for PwD for at least one year [37,38]. In addition, having a D or CD care dependency profile on the Katz-index scale Belgian version stands for having a diagnosis of dementia or having some characteristics linked to dementia, such as disorientation [39]. They must show CB, evaluated based on the Neuropsychiatric Inventory Questionnaire (NPI-Q). Caregivers answer this self-administered questionnaire; scores represent a sum of individual symptom scores ranging from 0 to 36 [40]. Palliative residents and residents who recently started a new medication were excluded.

### 2.4. Data Collection

Empirical data were collected using 24/7 participatory observations in the selected NH. Subjects were blinded unilaterally. Researchers explained to residents and staff how the study aims to map out what, on an average day, appears to prevent people from behaving desirably (e.g., actively making less noise, speaking less loudly, being more attentive to the environment). In each NH, the researchers observed the purposively selected people during three (eight hours) different time slots: 7:00 AM–3:00 PM, 3:00 PM–11:00 PM and 11:00 PM–7:00 AM. Through these three shifts, an observation of a full day (24 h) was ultimately performed. The observation was divided into three periods due to the feasibility and practicality of the observation. Eight hours is the maximum duration to ask a person to perform a participatory observation. In addition, to be able to blend in without interfering, the participatory observation followed the daily life of the NHs, which were divided into three shifts per day for nursing staff and personnel. The focus of the observations was on the key participants but also on the care providers and the residents around the key participants. 

The observations were held on the ward during all activities; eating, washing, resting, watching TV and when there was no activity. The observation protocol was based on the guidelines of participatory observation studies within an ethnographic design, as described by Dahlke [41]. The observation protocol was adapted to be applicable in people with dementia based on the long-time expertise with dementia of the healthcare professionals in the research group and was finally checked with the Ethical Committee. A characteristic of the participating observation (PO) is that the researcher participates in the same activities and blends in as a care team member. PO differs from naturalistic observation because the latter does not involve interaction between the researcher and participants. So, the two observers were present in the living area of the PwD in the NHs. They entered the observation area (e.g., the living room) in the morning by saying good morning and started helping with breakfast, just like the carers present at that moment. They mingled with the people present in the room, talking and when needed or asked, they also offered aid to go to the toilet or gave some water or something else, small tasks which are typically performed by carers. They were also wearing the same uniform as the carers. After some time, they left the living room to take notes of their observations [41].

Two researchers observed and collected the data; both were occupational therapists with extensive experience in NHs and working in the setting for some years. They were familiar with living, working, caring and interacting with the residents with dementia in NHs. They were not working in the NHs where they conducted the PO. Time-stamped observations were written out of sight of the key participants to avoid suspicion. The researcher described the situation and the environment, followed by the behavior, the incident, the interaction and how the participants responded. 

The observation methodology as described above was decided beforehand and was approved by the Ethical Committee. During the writing, attention was paid to writing literal, objective observations to obtain unbiased data. 

The observer took what are called field notes:

1. Field notes to include as much information as possible. Field notes are always recorded on site to ensure everything is captured (we made sure to finish the notes right after observation and before leaving the site. We were also writing notes throughout the observation). Including as much information as possible in the field notes were necessary;
2. Dates, times, and space identification;
3. Sensory of the space: light, sounds, smells, taste and texture of the material,
4. Any conversation or phrases used during the observation or phrases in any language or any insider conversation;
5. Any questions the observer may have concerning the site for further investigation;
6. Personal response to the facts that were observed,
7. All pages are numbered to keep the record in order,
8. For clarity, always keep four sections separated: notes, description of notes, analysis, and reflection of the observation.

The field notes are reconstructed at the end of each observation shift into a detailed transcript. A member check was carried out with employees or family; checks were made on whether the observations were correct and whether additional comments were needed. No audio/video recordings were used during observations.

### 2.5. Data Analysis

The collected data (detailed observation transcript) were used in an iterative process and constant comparison of the transcripts [42]. The data analysis followed a phenomenological–hermeneutical method in three phases: naïve understanding, structural analysis and comprehensive understanding. 

First, naïve understanding was formulated [43]. Then, for the structural analysis, researchers divided the transcripts into ‘meaning units’ according to the events. The Antecedent–Behavior–Consequence (ABC) model was used. Behavior was determined by a specific antecedent that happened before [44]. The consequence of the behavior was what happens afterwards, which was not always visible or observable as it can be a feeling (e.g., ‘feeling safe.’) Each meaning unit containing ABC was considered as one unit for analysis. When the transcript was divided into meaning units, some events occurred not as a consequence of the soundscape (e.g., apparent visual stimuli, pain, touching or a conversation); in these cases; the meaning unit was removed from the analysis. 

The next step in the structural analysis was condensing the meaning units; the essence of each ‘meaning unit’ was expressed as briefly as possible [43]. Two researchers identified the meaning units and the condensation of these units independently. The condensed meaning units were cross-validated via an iterative process between researchers to reach a consensus. The condensed meaning units were examined regarding similarities and differences. They were sorted, and similar condensed meaning units were abstracted to form subthemes. The research team constantly compared and discussed the condensed meaning units and subthemes to identify patterns, showing the relation between sound and behavior. The subthemes eventually were assembled into themes, which were then reflected regarding the naïve understanding. Although these steps are presented here linearly, these phases were characterized iteratively. Lastly, the themes and subthemes were reflected concerning the research question and the context and reported in a final synthesis.

### 2.6. Quality Insurance

Several quality criteria were considered in the procedure [34]. The researchers who performed the participatory observations were not employed in study wards to decrease bias (being too familiar or connected) and increase the trustworthiness of the data. The confirmability was reinforced by taking field notes and supplementing observations based on a member check. To increase the credibility, two researchers condensed the meaningful units independently and performed a peer debriefing of the different steps throughout the analysis. Inclusion and exclusion criteria for the sample and a detailed description of the characteristics of the applied example increased transferability. As a result, adequate information was noted, making the result transferable to other contexts. Therefore, other researchers can use the outcome.

## 3. Results

Thirty-five key participants were included in the final sample, residing in nine NHs and observed during the 24 h observation. During the observations, field notes were written, and a transcript was generated. Table 1 shows the main characters displayed per resident and the sample description.

### 3.1. Naïve Understanding

During the day, the living space was filled with the sound of the radio or TV, but the soundscapes were experienced as monotonous. The hallways and bedrooms were quiet. The PwD were resting or wandering around without meeting each other, resulting in little to no sound, and this added to the already discovered fact that there were no statistical differences regarding sound levels in the different nursing homes. However, there was much noise during mealtime, and the acoustic environment was hectic and heterogeneous. All the PwD and (professional) caregivers were in one area. The sound of human voices, cutlery, crockery and plates being stacked or passed around dominated the acoustic environment. At night, the acoustic environment was silent. When caregivers passed by for check-ups, there was a brief burst of noise, the sound of closing doors and rolling carts.

“It has been observed that PwD has an inadequate reaction to the sound, which was described as either not understanding the sound’s meaning, the source of the sound and the reason behind it.” It was observed that PwD reacted to sound and the absence of sound. When PwD were overstimulated with auditory stimuli, they tended to leave the noisy space—probably—to feel safe. When they could not flee, they became nervous and angry. It was also observed that when there were not enough auditory stimuli, PwD became scared. They tended to start wandering around or creating stimuli by talking, making noises or manipulating objects.

### 3.2. Structural Analysis

The structural analysis resulted in 125 meaning units. Table 2 shows five meaning units to illustrate how the transcript was divided, structured and formulated themes.

#### 3.2.1. Theme 1: Acoustic Can Prevent the Onset of CB

When the sound was familiar, the resident’s reaction appeared predictable, triggering less CB; strange or unfamiliar sounds evoked CB.

*“Seven residents are sitting around the table in the kitchen. The doorbell rings: it sounds throughout the building. Resident 7 gets up and walks towards the corridor.”*

Further analysis showed how residents endured complex sonic environments around loved ones. They seemed to feel safe and showed no CB.

*“Resident 7 is seated with another resident, whom he considers his wife. Another resident comes along, talking and humming; a caregiver walks by with rattling keys. The footsteps of the caregiver are audible. A TV and music can be heard in the distance. Resident 7 murmurs calmly to the resident with whom he is holding hands.”*

#### 3.2.2. Theme 2: The Absence of Acoustic Triggers Causes Anxiousness and Mistrust. Wandering, Talking or Manipulating Objects Can Create a Feeling of Safety

The ward could be quiet or monotonous during the day; for example, after breakfast, when people finished eating and tables were cleared. During these periods, residents rested or sat in the living room or a sitting area. Different forms of behavior were observed, seemingly to create a sense of safety. It appeared that residents had an overall feeling of being unsafe and showed fear or mistrust if insufficient stimuli were present. Some of them were humming, singing or talking to themselves or people passing by, not necessarily having a proper conversation to break the silence.

*“The hallway is empty and quiet. The night shift cart is being driven around; the wheels run on the floor tiles. The caregivers whisper and enter the room as quietly as possible. Resident 5 sits on his chair in his room, talking to himself.”*

It was also observed that some residents started manipulating objects such as sofas, garbage bins, gloveboxes or closed doors. They moved the objects, tried to open them, and played or tinkered with them. Their goal may be to create more stimuli.

*“It’s night, all the residents are in bed, and the ward is quiet. The radio is off. Resident 7 is standing in the kitchen, turning the knobs on the radio. He keeps doing this for 20 min.”*

When the residents had nothing to do, they wandered around. Some residents stepped towards the sound, possibly to look for auditory stimuli. Other residents continued to wander around, walking up and down the corridors or going back and forth between the sitting room, dining room and their rooms.

*“The television is on in the dining room. The TV is very loud. Resident 1 comes out of the silent corridor through the door and walks halfway in the room while watching the TV; she stays halfway through the room and continues watching the TV from a distance.”*

For residents unable to walk or move independently, they continuously tried to leave the room by, for example, trying to get out of their wheelchairs.

#### 3.2.3. Theme 3: Complex Sound Environments Cause an Uncomfortable or Angry Feeling, Which Can Be Solved by the Resident’s Behavior That Set Outs to Avoid or Reduce the Noise

On the other end of the spectrum, with various noises, the residents felt overstimulated. When the acoustics were loud and heterogeneous, it was observed as creating an uncomfortable feeling of suspicion; it made residents restless or angry.

*“Resident 2 is in the dining room, back-to-back with resident 1. Resident 1 talks a lot, loud and well audible; she looks angry over her shoulder several times when resident 1 talks. In the meantime, classical music playing non-dominant.”*

The residents tried eliminating the extra sound. Some residents would leave the room and look for a quiet place. Those wandering around tended to avoid the overly crowded and loud rooms by turning around, bypassing and walking toward other spaces.

*“Resident 14 sits at the table in the dining room. The food is served. There is the clatter of cutlery. Staff and volunteers walk around; people talk at the tables. Resident 14 gets up and walks out of the dining area.”*

However, not all people for whom the space was noisy would leave the room. Some would stay, angry and frustrated, not knowing how to act to reduce agitation—others, not physically able to move, kept trying.

*“After dinner, resident 8 sits at the table in his wheelchair, fixated with a belt around the waist. There is much talking and singing. Resident 8 constantly tries to stand up straight from his wheelchair, despite the fixation. Resident 14 is taken out of the bath and dressed; the caretaker starts blow-drying. Resident 14 sits on a chair and allows this but puts the fingers in the ears, holding the head down.”*

#### 3.2.4. Theme 4: PwD Can Misinterpret Sounds and Therefore Do Not React in a Way That Is Expected

Determining the sound source and understanding that there was no danger was usually enough for residents to feel safe and pursue what they were doing before hearing the sound.

*“Resident 7 sits in the seats in the hallway with his eyes closed. Ward is quiet. A laptop makes noise. Resident 7 looks up in the direction of the laptop, then looks back in front of him and closes his eyes.”*

If residents did not recognize or understand the sound, they reacted unexpectedly by yelling in the direction of the sound, laughing (to the sound of broken glass) or dancing (to a ringing phone). Some residents shook their heads or raised their eyebrows at sounds they did not comprehend.

*“Resident 10 gets up from the toilet. The caregiver flushes without announcing it. Resident 10 reacts by saying: “Oh God, who was that?”*

The resident could be scared when the caregiver makes a sound without announcing it. When the resident understood the sound and the expectation, they could react as expected. e.g., going to the door when the bell rings or whispering when someone nearby is on the phone.

*“All seven residents sit around the table in the kitchen. The doorbell rings (sound is audible throughout the building). Resident 7 stands up and walks towards the corridor.”*

### 3.3. Comprehensive Understanding

PwD had complex reactions to the soundscape, with the onset of CB dependent on whether residents felt safe. When they felt threatened, CB occurred, although the unsafe feeling could have different roots. CB appeared to be triggered by an excess or lack of stimuli, which was highly personal and depended on personality or the time of day. In addition, the nature of the stimuli, familiar or unknown, was a determining factor for the onset and progression of CB. Familiar sounds, the amount tuned to the capacity of the PwD, could reduce CB. Finally, the presence of significant others could influence the reaction to the acoustic environment, possibly by increasing the feeling of safety. 

**Figure 1:**
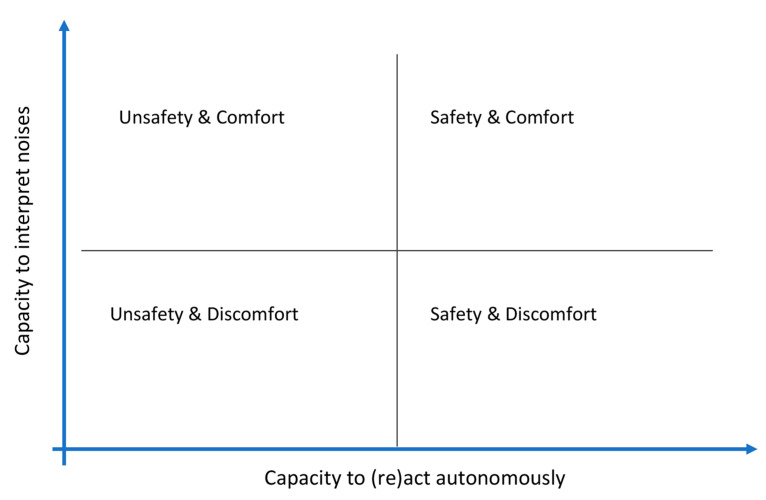
The four personae derived from the data show the interaction between the person’s capacity to interpret sounds and their capacity to react to them.

In conclusion, the emergence and progression of CB are highly individual (relying on personality and characteristics of dementia) and depend on the interaction between persons and the acoustic environment. The influence of sounds on the behavior and QoL of PwD was subject to two dimensions: (1) the ability to correctly interpret the sound and (2) the ability to react to it adequately. These two dimensions were interrelated and led to four different types: the PwD (1) who can interpret correctly and react adequately, (2) who can interpret correctly but cannot react adequately, (3) who cannot interpret correctly but reacts adequately and (4) who cannot interpret correctly and cannot react adequately. Four personae emerged from the qualitative data (Figure 1), offering opportunities to understand the PwD’s reactions to the sonic environment and develop adequate soundscapes.

## 4. Discussion

This study aimed to examine the influence of acoustic and acoustical triggers on CB. CB is seen as an active attempt to articulate an unmet need and is a pervasive problem in PwD [4]. It burdens caregivers, reduces QoL and increases the risk of institutionalization [2,13]. Therefore, it is crucial to reduce CB. 

Managing CB takes work. The antipsychotic practice guideline in care homes formed recommendations concerning antipsychotics as a treatment for CB, but not as the first approach. Non-pharmacological interventions should be tried first; if antipsychotics are used, they must be combined with non-pharmacological interventions [3,7,45]. Understanding the reason behind the behavior is essential in managing CB non-pharmacologically [14,46]. Research into influencing factors leading to CB shows the effects of physical, psychological, communicational, social and environmental factors [4,47]. Knowing these factors can create the opportunity to adapt and prevent thereby reducing CB. Much research has looked at non-pharmacological approaches, such as physical exercise, animal-assisted therapy or touch therapy [6,16]. Nonetheless, they are often individual and time-consuming. Therefore, positively adapting the environment to influence CB is interesting. 

Adapting the environment is already a topic of interest in the literature. Approaches such as light therapy or aromatherapy have been intensively researched [6,16]. Sound has also been the subject of examination. However, sound research is often about music therapy [48,49] or musical background [49]. In recent years, acoustic and soundscapes received increasing interest in research within various target groups, such as secondary school pupils [19], people with profound intellectual and multiple disabilities [50] or PwD [29,31,51].

In a soundscape study, the first step is to view each person individually and map out the sounds that make them feel safe, considering what is recognizable and familiar to the person. Awareness of the specific soundscape and knowing the resident’s background and interests enable the caregivers to influence this actively and design the acoustic environment to the needs of PwD. That means that as a health care professional, one should try to complete an acoustical anamnesis to detect what the familiar sounds are for the person. This can be performed, e.g., by asking the proxies and other family members about the sounds the PwD have been exposed to during their life span.

The observations show that CB seems to be influenced by an excess or lack of auditory stimuli, which is very personal. CB diminishes if the number of auditive stimuli can be adapted and if the PwD feels safe. The feeling of safety is created by familiar and comprehensible sounds and being around trusted people. Therefore, personal, tailored intervention [52] is the key to having a positive soundscape, as the effect can vary between people but also within one person based on different times of the day, personality and familiarity and recognition of the sound. 

Possible explanations for these results are the ‘ecological equation’ of Kurt Lewin (cited in Lawton, 1977) and the ‘ecological model of aging’ of Lawton [53]. Lewin states that behavior results from people and environments. These transactional models, such as the Person Environment Fit model [54] and the Ecology of Human Performance framework [55], support this concept stating that people cannot be seen separately from their environment with continuous interaction between the two. This connection is consistent with the finding that CB is a consequence of the person’s environment. The relation with the environment corresponds with the work of Gerritsen et al. [46] that notes that CB is not a direct symptom of dementia but rather an indirect consequence of something possibly present in the environment. CB functions as a signal that well-being is threatened. 

In addition to the ‘ecological equation’ to comprehend the interaction between person and environment, Lawton [53] designed an ecological model of aging, which showed that behavior is a function of the individual’s competence or capacity and the situation’s environmental press. The environmental press corresponds to the demand the environment imposes on individuals and can be behavior-activating to some [53]. Adaptation to environmental stressors may depend on the individual’s level of competence [56]. Various situations with various levels of press can have multiple behavioral outcomes for people. The same result was also concluded from the observations. The capacity of the PwD can change during different moments, where, e.g., the PwD can show more CB in evenings compared to mornings, although the acoustic environment is similar. Krishnamoorthy and Anderson [4] discuss the lack of stimulation as a possible reason for wandering. Tible et al. [47] also address the optimization of stimulation levels, considering an individual’s capacity. The optimization of this level of stimulation is seen as an environmental characteristic related to lower levels of CB.

Krishnamoorthy and Anderson [4] briefly describe that persistent noise causes stress and annoyance. This description corresponds to the fact that, e.g., the PwD wants to leave the room or become agitated while sitting and eating their meal in a noisy room. They also state that a familiar space can reduce CB [4], which can explain the observation of reduced CB when being around familiar people. Recognizability and familiarity create a safe feeling, making the CB less present.

This participatory observational study has some strengths, for example, the sample was substantial. Thirty-five PwD were observed in nine different NHs and at various times throughout the day, resulting in 24 h of observation (420 h total). This amount of data ensured that the collected data were pervasive and varied. 

Researching the influence of the acoustic environment on CB to create a basis for a soundscape is innovative, as sound has only been studied to a limited extent. Qualitative research was the best way to surface these insights and form a clear understanding of the influences.

The analysis was structured using the ABC method, developing the separated meaning units and then condensing the meaning units to form subthemes and themes. Based on peer debriefing among the different researchers, the condensed meaning units, subthemes, and themes were cross-validated, increasing the credibility. Because of the detailed description of the residents included in the sample, the results can easily apply to one’s own sample, and transferability is increased. 

This study also has some limitations. The 24 h observations were split into three timeslots, performed on different days. Therefore, some data could be missed, or links between behavior and earlier events could be overlooked. It is, however, impossible to be present for 24 consecutive hours. No video was recorded, making it impossible to check to confirm or complete incomplete field notes. Due to the dynamic nature of the observations, it would be challenging to record; additionally, it would not be ethical to record PwD’s CB. To improve confirmability, the researcher checked with care providers who worked during the shift when the field notes were incomplete. 

The participants were not subjected to an audiometric test. In fact, an audiometric test would be sufficient; however, in the case of PwD, the test is not possible. In addition, the research team did not want to interfere with residents’ daily life. The electronic patient record and nursing report were checked to understand the participant’s hearing situation (in accordance with the study’s ethical approval).

During the observations, it was not always apparent whether CB occurred due to the acoustic environment. It was observed that some residents started manipulating objects. They moved the objects, tried to open them and played or tinkered with them. By doing so, they may want to create more stimuli. We do not know if the lack of stimuli makes them start manipulating or if the sight of the object lures them to manipulate them. It can, however, be seen as aberrant motor behavior, a form of CB [57]. It is also impossible to eliminate every other stimulus, such as a visual stimulus or the experience of pain, which are also proven to influence CB [4]. One solution is to design an experimental study; however, it is unethical to provoke CB by creating too many stimuli or leaving the PwD under-stimulated. Nevertheless, this uncertainty has been considered during the meaning unit’s inclusion. The meaning unit was omitted when there was doubt about the trigger. 

The conclusion of being able to cope better in a complex sound environment when a significant other is around was only observed in one participant. Due to the COVID-19 outbreak, it was impossible to include more participants to reach saturation. It could be the subject of future research to validate this finding. In addition, it could be studied as to whether high staff turnover (constantly being cared for by new, unfamiliar caregivers) influences the feeling of safety and thus affects the onset of CB. 

It is beyond the scope of this study to map out the ideal type of sound since no sounds were created during the observations, and the sonic environment was seen holistically. During the structural analysis, it was concluded that familiar or recognizable sounds (such as a ringing doorbell or talking) could create more peace than unknown or unrecognizable sounds (a plane flying over). Nevertheless, acknowledging the underlying “aural diversity”, i.e., the reality of everyone’s different experience of hearing and understanding of the soundscapes [58], is the first step toward designing inclusive and supportive acoustic environments for PwD. Further research is needed to explore the ratios of different sounds, sound volume and their influence on CB, and an individual’s capacity to create a soundscape suited for the resident. It can also be interesting to study acoustic comfort in NHs using deaf and hard-of-hearing PwD. Wiratha and Tsaih [59] assessed this based on normal-hearing individuals and concluded that sounds of nature contributed to a positive impression of the acoustic environment. Aletta et al. [33] mapped out the holistic perception of the sound environment in the NH. Erfanian et al. [60] looked at how the perception of the acoustic environment correlates with the physiological properties stimulated by the soundscape.

## 5. Conclusions

Although sonic interventions, such as music therapy and the addition of classical music, have been implemented, soundscape research is rare in NHs. In a soundscape study, the first step is to view each person individually and map out the sounds that make them feel safe, considering what is recognizable and familiar to the person. Awareness of the specific soundscape and knowing the resident’s background and interests enable the caregivers to influence this actively and design the acoustic environment to the needs of PwD.

Based on this study’s findings, working with familiar and recognizable sounds is essential when soundscapes are created to improve the quality of care and reduce BPSD. Therefore, soundscape can function as a non-pharmacological approach to reduce CB. It was observed that residents showed less CB when the sounds were expected by announcing them or when people could identify the source of the sound by looking up and giving the sound meaning. Therefore, it is advisable to notify residents of each action during the care instead of alarming them with unexpected noise.

In conclusion, essential aspects of the preferred soundscape were discovered. Further research into which sounds are familiar and the capacity of a PwD is necessary to optimize the development of soundscapes.

This participatory observational study relied on a substantial sample for qualitative research and was based on a strong method using 24-h observations in three timeslots. That increased the credibility of the results. Video recording could have helped us to complete field notes, but we decided not to do this based on ethical issues.

This study and the resulting personae helped us design a soundscape based on PwD’s needs and to reduce BPSD in dementia. (e.g., reduce sounds that create fears due to misinterpretation and replace them with familiar sounds). The results also helped overall soundscape design in dementia care; the idea of a healthy and healing environment focused on light, temperature and smell can now have soundscape as an addition.

## Figures and Tables

**Table 1:** The data collection set out to include two waves of NHs. Table 1 shows the main characters displayed per resident and presents the description of the sample.

Wave 1					
Resident	Nursing Home	Sex	Katz-Scale Score	MMSE	NPI-Q
1	1	Female	D	/ ^(1)^	16/36
2	1	Female	CD	2/30	8/36
3	1	Female	CD	2/30	13/36
4	1	Female	D	8/30	10/36
5	1	Male	D	/ ^(2)^	20/36
6	2	Female	D	7/30	6/36
7	2	Male	D	6/30	8/36
8	2	Male	CD	/ ^(1)^	5/36
9	2	Female	D	/ ^(1)^	5/36
10	2	Female	D	5/30	8/36
11	3	Male	D	14/30	15/36
12	3	Female	CD	10/30	10/36
13	3	Female	CD	13/30	11/36
14	3	Female	CD	2/30	12/36
15	3	Female	D	/ ^(2)^	9/36
16	4	Female	D	11/30	8/36
17	4	Male	CD	4/30	10/36
18	4	Female	CD	/ ^(1)^	
19	4	Female	CD	8/30	10/36
20	4	Female	D	/ ^(2)^	20/36
**Wave 2**					
**Resident**	**Nursing Home**	**Sex**	**Katz-Scale Score**	**MMSE**	**GDS Score**
1	1	Female	CD	8/30	7
2	1	Male	CD	/	6
3	1	Female	D	/	6
4	2	Female	CD	/	6
5	2	Male	CD	/	6
6	2	Male	CD	/	5
7	3	Female	CD	/	7
8	3	Female	CD	16/30	5
9	3	Male	CD	12/30	6
10	4	Female	CD	/	7
11	4	Female	CD	/	6
12	4	Female	B	/	4
13	5	Female	B	15/30	5
14	5	Female	B	1/30	5
15	5	Male	B	6/30	6

^(1)^ Not possible to take the test; ^(2)^ Refused to take the test.

**Table 2:** Five meaning units of structural analysis.

Meaning Unit			Condensation	Subtheme	Theme
Antecedent	Behaviour	Consequence			
The caregiver goes to the wardrobe (which is against the outside wall of the bathroom). She opens the wardrobe door with a key (it makes a lot of noise). The doors are closed again, making a loud slamming noise. The room was completely silent, making the sound very noticeable	Resident 1 yells, while standing in the bathroom: boo boo!	The caregiver yells back at her: “It’s okay, I am just messing around in the wardrobe.”	Yelling in response to a striking sound that is very noticeable in a completely silent space	Vocal reaction to sound that is not understood	Not understanding the sound
Resident 14 sits at the table in the dining room. The food is scooped out. There is the clatter of cutlery. People (staff and volunteers) walk around the room and provide everyone with a plate of food. There is talk at the tables	Resident 14 gets up and walks out of the dining area towards the hallway	The caregiver asks her: “Wouldn’t it be better to stay seated? Your food will be served in a minute.”	Getting up, leaving the dining room, which is very crowded with voices and the cutlery	Moving away from a noisy and crowded space	Too many stimuli
Resident 17 sits in the dining/living room at a table. Twelve other residents are in the room, four aid workers and a family member. The family member talks to a resident at the table next to resident 17. The care workers stand behind him, talking. Sometimes the cat meows. You can hear spoons sounding against porcelain.	Resident 17 eats soup and looks over his shoulder at where the care workers are talking	He starts talking to the residents at his table. He talks to himself but directs it to others at the table	Looking over the shoulder in the direction of the voice and then talking to oneself and people at the table in a room with a lot of ambient noise.	Looking in the direction of the sound. After determining he is safe, resume activity.	Identifying the sound
In the living room, the TV and radio are on simultaneously. The robocat is making a purring sound on the table	Resident 16 talks in the cat’s ear and says, “So sweet; you’re so sweet.”	/	Calm and sweet talking to the robocat.	Calmly talking	No CB
The hallway is quiet. The sound of the dining area is occasionally audible in the distance. In some parts of the corridor, it is not audible at all.	She begins to walk down the corridor (these are endless, arranged in a square). She talks to herself and walks down the corridor.	She keeps walking around and talking to herself.	Wandering around in a very quiet hallway and talking to oneself.	Wandering around because of under-stimulation	Not enough stimuli

## Data Availability

Data are available upon request; please note that data are in Dutch and not English.

## References

[1] World Health Organization (2018). Integrated Care for Older People: Realigning Primary Health Care to Respond to Population Ageing.

[2] Finkel S. (2000). Introduction to behavioural and psychological symptoms of dementia (BPSD). Int. J. Geriatr. Psychiatry.

[3] Azerma M. (2015). Dealing with behavioral and psychological symptoms of dementia: A general overview. Psychol. Res. Behav. Manag..

[4] Krishnamoorthy A., Anderson D. (2011). Managing challenging behaviour in older adults with dementia. Prog. Neurol. Psychiatry.

[5] Benoit M., Arbus C., Blanchard F., Camus V. (2006). Professional consensus on the treatment of agitation, aggressive behaviour, oppositional behaviour and psychotic disturbances in dementia. J. Nutr. Health Aging.

[6] Oliveira A.M.D., Radanovic M., Mello P.C.H.D., Buchain P.C., Vizzotto A.D.B., Celestino D.L., Stella F., Piersol C.V., Forlenza O.V. (2015). Nonpharmacological Interventions to Reduce Behavioral and Psychological Symptoms of Dementia: A Systematic Review. Biomed. Res. Int..

[7] Liperoti R., Pedone C., Corsonello A. (2008). Antipsychotics for the Treatment of Behavioral and Psychological Symptoms of Dementia (BPSD). Curr. Neuropharmacol..

[8] Ricci G. (2019). Social Aspects of Dementia Prevention from a Worldwide to National Perspective: A Review on the International Situation and the Example of Italy. Behav. Neurol..

[9] Agüero-Torres H. (2001). Institutionalization in the elderly The role of chronic diseases and dementia. Cross-sectional and longitudinal data from a population-based study. J. Clin. Epidemiol..

[10] Fauth E.B., Gibbons A. (2014). Which behavioral and psychological symptoms of dementia are the most problematic? Variability by prevalence, intensity, distress ratings, and associations with caregiver depressive symptoms. Int. J. Geriatr. Psychiatry.

[11] Hurt C., Bhattacharyya S., Burns A., Camus V., Liperoti R., Marriott A., Nobili F., Robert P., Tsolaki M., Vellas B. (2008). Patient and Caregiver Perspectives of Quality of Life in Dementia. Dement. Geriatr. Cogn. Disord..

[12] Samus Q.M., Rosenblatt A., Steele C., Baker A., Harper M., Brandt J., Mayer L., Rabins P.V., Lyketsos C.G. (2005). The Association of Neuropsychiatric Symptoms and Environment with Quality of Life in Assisted Living Residents With Dementia. Gerontologist.

[13] Feast A., Moniz-Cook E., Stoner C., Charlesworth G., Orrell M. (2016). A systematic review of the relationship between behavioral and psychological symptoms (BPSD) and caregiver well-being. Int. Psychogeriatr..

[14] Cook E.D.M., Swift K., James I., Malouf R., de Vugt M., Verhey F. (2012). Functional analysis-based interventions for challenging behaviour in dementia. Cochrane Database Syst. Rev..

[15] O’Neil M., Freeman M., Christensen V., Telerant R., Addleman A., Kansagara D. (2011). A Systematic Evidence Review of Non-pharmacological Interventions for Behavioral Symptoms of Dementia.

[16] Abraha I., Rimland J.M., Trotta F.M., Dell’Aquila G., Cruz-Jentoft A., Petrovic M., Gudmundsson A., Soiza R., O’Mahony D., Guaita A. (2017). Systematic review of systematic reviews of non-pharmacological interventions to treat behavioural disturbances in older patients with dementia. The SENATOR-OnTop series. BMJ Open.

[17] Dyer S.M., Harrison S.L., Laver K., Whitehead C., Crotty M. (2018). An overview of systematic reviews of pharmacological and non-pharmacological interventions for the treatment of behavioral and psychological symptoms of dementia. Int. Psychogeriatr..

[18] Bourdon E., Havreng-Théry C., Lafuente C., Belmin J. (2022). Effect of the Physical Environment on Health and Well-Being of Nursing Homes Residents: A Scoping Review. J. Am. Med. Dir. Assoc..

[19] Connolly D., Dockrell J., Shield B., Conetta R., Mydlarz C., Cox T. (2019). The effects of classroom noise on the reading comprehension of adolescents. J. Acoust. Soc. Am..

[20] Wilson J.D., McGinnis N., Latkova P., Tierney P., Yoshino A. (2016). Urban Park Soundscapes: Association of Noise and Danger with Perceived Restoration. J. Park Recreat. Adm..

[21] Brown B., Rutherford P., Crawford P. (2015). The role of noise in clinical environments with particular reference to mental health care: A narrative review. Int. J. Nurs. Stud..

[22] Andringa T., Lanser J. (2013). How Pleasant Sounds Promote and Annoying Sounds Impede Health: A Cognitive Approach. Int. J. Env. Res. Public Health.

[23] Truax B. (1984). Acoustic Communication.

[24] (2014). Acoustics-Soundscape-Part 1: Definition and Conceptual Framework. Acoustique-Paysage Sonore-Partie 1: Définition et Cadre Conceptuel.

[25] Axelsson Ö., Nilsson M.E., Berglund B. (2010). A principal components model of soundscape perception. J. Acoust. Soc. Am..

[26] Aletta F., Botteldooren D., Thomas P., Vander Mynsbrugge T., De Vriendt P., Van de Velde D., Devos P. (2017). Monitoring sound levels and soundscape quality in the living rooms of nursing homes: A case study in Flanders (Belgium). Appl. Sci..

[27] Van den Bosch K.A.M., Welch D., Andringa T.C. (2018). The evolution of soundscape appraisal through enactive cognition. Front. Psychol..

[28] Thomas P., Aletta F., Filipan K., Vander Mynsbrugge T., De Geetere L., Dijckmans A., Botteldooren D., Petrovic M., Van de Velde D., De Vriendt P. (2020). Noise environments in nursing homes: An overview of the literature and a case study in Flanders with quantitative and qualitative methods. Appl. Acoust..

[29] Devos P., Thomas P., Aletta F., Vander Mynsbrugge T., De Vriendt P., Van de Velde D., Botteldooren D. Towards Understanding Healthy and Supportive Acoustic Environments: The case of a nursing home. Proceedings of the International Congress on Acoustics.

[30] van den Bosch K.A., Andringa T.C., Post W.J., Ruijssenaars W.A.J.J.M., Vlaskamp C. (2018). The relationship between soundscapes and challenging behavior: A small-scale intervention study in a healthcare organization for individuals with severe or profound intellectual disabilities. Build. Acoust..

[31] De Pessemier T., Vanhecke K., Thomas P., Vander Mynsbrugge T., Vercoutere S., Van de Velde D., De Vriendt P., Joseph W., Martens L., Botteldooren D. (2022). Personalising augmented soundscapes for supporting persons with dementia. Multimed. Tools Appl..

[32] Kosters J., Janus S.I.M., van den Bosch K.A., Zuidema S., Luijendijk H.J., Andringa T.C. (2022). Soundscape Optimization in Nursing Homes Through Raising Awareness in Nursing Staff WITH MoSART+. Front. Psychol..

[33] Devos P., Aletta F., Vander Mynsbrugge T., Thomas P., Filipan K., Petrovic M., De Vriendt P., Van de Velde D., Botteldooren D. (2018). Soundscape design for management of behavioral disorders: A pilot study among nursing home residents with dementia. INTER-NOISE and NOISE-CON Congress and Conference Proceedings.

[34] Portney L.G., Watkins M.P. (2007). Foundations of Clinical Research: Applications to Practice.

[35] Agentschap Zorg en Gezondheid (2017). Besluit van de Vlaamse Regering tot Wijziging van Bijlage XII Bij het Besluit van de Vlaamse Regering van 24 Juli 2009 Betreffende de Programmatie, de Erkenningsvoorwaarden en de Subsidieregeling Voor Woonvoorzieningen en Verenigingen van Gebruikers en Mantelzorgers, wat de Voorwaarden Infrastructuur Betreft. https://codex.vlaanderen.be/PrintDocument.ashx?id=1032439&datum=&geannoteerd=false&print=false.

[36] Patton M.Q. (2014). Qualitative Research & Evaluation Methods: Integrating Theory and Practice.

[37] Folstein M.F., Folstein S.E., McHugh P.R. (1975). Mini-mental state. J. Psychiatr. Res..

[38] Kok R., Verhey F. (2002). Dutch translation of the Mini Mental State Examination.

[39] Katz S. (1963). Studies of Illness in the Aged. JAMA.

[40] Kaufer D.I., Cummings J.L., Ketchel P., Smith V., MacMillan A., Shelley T., Lopez O.L., DeKosky S.T. (2000). Validation of the NPI-Q, a Brief Clinical Form of the Neuropsychiatric Inventory. J. Neuropsychiatry Clin. Neurosci..

[41] Dahlke S., Hall W., Phinney A. (2015). Maximizing theoretical contributions of participant observation while managing challenges. Qual. Health Res..

[42] Taylor R.R. (2017). Kielhofner’s Research in Occupational Therapy: Methods of Inquiry for Enhancing Practice.

[43] Lindseth A., Norberg A. (2004). A phenomenological hermeneutical method for researching lived experience. Scand. J. Caring Sci..

[44] Volicer L., Hurley A.C. (2003). Review Article: Management of Behavioral Symptoms in Progressive Degenerative Dementias. J. Gerontol. A Biol. Sci. Med. Sci..

[45] Zuidema S.U., Johansson A., Selbaek G., Murray M., Burns A., Ballard C., Koopmans R.T. (2015). A consensus guideline for antipsychotic drug use for dementia in care homes. Bridging the gap between scientific evidence and clinical practice. Int. Psychogeriatr..

[46] Gerritsen D.L., Smalbrugge M., Veldwijk-Rouwenhorst A.E., Wetzels R., Zuidema S.U., Koopmans R.T.C.M. (2019). The Difficulty with Studying Challenging Behavior. J. Am. Med. Dir. Assoc..

[47] Tible O.P., Riese F., Savaskan E., von Gunten A. (2017). Best practice in the management of behavioural and psychological symptoms of dementia. Adv. Neurol. Disord..

[48] Wang G., Albayrak A., van der Cammen T.J.M. (2019). A systematic review of non-pharmacological interventions for BPSD in nursing home residents with dementia: From a perspective of ergonomics. Int. Psychogeriatr..

[49] Chaudhury H., Hung L., Badger M. (2013). The Role of Physical Environment in Supporting Person-centered Dining in Long-Term Care. Am. J. Alzheimer’s Dis. Other Dement..

[50] van den Bosch K.A., Andringa T.C., Başkent D., Vlaskamp C. (2016). The Role of Sound in Residential Facilities for People with Profound Intellectual and Multiple Disabilities. J. Policy Pr. Intellect. Disabil..

[51] Aletta F., Vander Mynsbrugge T., Van de Velde D., De Vriendt P., Thomas P., Filipan K., Botteldooren D., Devos P. (2018). Awareness of ‘sound’ in nursing homes: A large-scale soundscape survey in Flanders (Belgium). Build. Acoust..

[52] Koch J., Amos J.G., Beattie E., Lautenschlager N.T., Doyle C., Anstey K.J., Mortby M.E. (2022). Non-pharmacological interventions for neuropsychiatric symptoms of dementia in residential aged care settings: An umbrella review. Int. J. Nurs. Stud..

[53] Lawton M.P. (1977). An Ecological Theory of Aging Applied to Elderly Housing. J. Archit. Educ..

[54] Law M., Cooper B., Strong S., Stewart D., Rigby P., Letts L. (1996). The Person-Environment-Occupation Model: A Transactive Approach to Occupational Performance. Can. J. Occup. Ther..

[55] Turpin M., Iwama M. (2010). Using Occupational Therapy Models in Practice.

[56] Lawton M.P. (1985). The Elderly in Context. Env. Behav..

[57] Zhao Q.F., Tan L., Wang H.F., Jiang T., Tan M.S., Tan L., Xu W., Li J.Q., Wang J., Lai T.J. (2016). The prevalence of neuropsychiatric symptoms in Alzheimer’s disease: Systematic review and meta-analysis. J. Affect. Disord..

[58] Drever J.L., Hugill A. (2022). Aural Diversity.

[59] Wiratha M.S., Tsaih L. (2015). Acoustic comfort in long-term care facilities based on listening impressions from normal hearing individuals. Proc. Meet. Acoust..

[60] Erfanian M., Mitchell A.J., Kang J., Aletta F. (2019). The Psychophysiological Implications of Soundscape: A Systematic Review of Empirical Literature and a Research Agenda. Int. J. Env. Res. Public Health.

